# Highly Stable Flexible SERS-Imprinted Membrane Based on Plasmonic MOF Material for the Selective Detection of Chrysoidin in Environmental Water

**DOI:** 10.3390/polym17010081

**Published:** 2024-12-31

**Authors:** Xinyi Liu, Hongji Li, Dandan Wang, Jian Lu, Yilin Wu, Wei Sun

**Affiliations:** 1 Hainan Engineering Research Center of Tropical Ocean Advanced Opto-Electrical Functional Materials, College of Chemistry and Chemical Engineering, Hainan Normal University, Haikou 571158, China; 2022101447@mails.cust.edu.cn (X.L.); sunwei@hainnu.edu.cn (W.S.); 2 Key Laboratory of Advanced Materials of Tropical Island Resources, Ministry of Education, School of Chemistry and Chemical Engineering, Hainan University, Haikou 570228, China; 3 School of Fashion and Textiles, The Hong Kong Polytechnic University, Hong Kong 999077, China; jian.lu@polyu.edu.hk; 4 Institute of Green Chemistry and Chemical Technology, Advanced Chemical Engineering Laboratory of Green Materials and Energy of Jiangsu Province, School of Chemistry and Chemical Engineering, Jiangsu University, Zhenjiang 212013, China; wuyilin@ujs.edu.cn

**Keywords:** surface-enhanced Raman scattering, molecular imprinting technology, plasmonic MOF material, selective detection, organic dye

## Abstract

Chrysoidin (CG) can be ingested into the human body through the skin and cause chronic toxicity, so the detection of CG levels in the environment is crucial. In this study, we synthesize F-Ag@ZIF-8/PVC molecular-imprinted membranes (FZAP-MIM) by an innovative combination of SERS detection, membrane separation, and a molecular-imprinted technique in order to perform the analysis of CG in water. The plasmonic MOF material as a SERS substrate helps to enrich the target and realize the spatial overlap of the target with the nanoparticle tip “hotspot”. To avoid the poor reproducibility of Raman signals caused by the random arrangement of the powder substrate, polyvinyl chloride (PVC) is used to provide support and protection for the powder substrate. PVC has excellent dirt immunity and chemical stability, enabling the substrate to maintain Raman performance under complex and extreme detection conditions. FAZP-MIM has outstanding sensitivity and selectivity and can quickly and accurately capture targets even in the presence of similar structural interferences. The method showed superior recoveries in spiked recovery tests of real water samples and is expected to be practically applied to the trace detection of organic dye molecules in the environment.

## 1. Introduction

Chrysoidin (CG) is a synthetic azo dye mainly used in the textile industry, containing azo groups and aromatic rings, which can cause chronic poisoning when accidentally ingested or absorbed through the skin [1,2]. Therefore, it is necessary to develop a rapid and efficient method for the detection of organic dyes in the environment. Conventional methods used to date for the detection of CG include high-performance liquid chromatography (HPLC) [3], liquid chromatography–mass spectrometry (LC-MS) [4], and thin-layer chromatography (TLC) [5], and the cumbersome nature of the detection process has limited their application.

The surface-enhanced Raman scattering (SERS) technique is a highly sensitive, fast-responding, non-destructive analytical tool [6] that has a promising application in the trace detection of ultra-low concentration analytes. The enhancement mechanism of SERS mainly originates from the localized surface plasmon resonance (LSPR) of metallic nanostructures, which generates a dramatic localized electromagnetic enhancement (EM) and amplifies the inelastic Raman scattering signals of the target molecules [7]. Plasmonic MOF materials are nanostructures combining noble metal nanoparticles and organic porous materials that exhibit excellent optical properties in surface plasma correlation fields [8]. Since plasmonic MOF materials have a strong electromagnetic field and a high density of reaction sites, they have shown significant potential in the field of SERS detection in recent years [9,10]. Since LSPR depends on the surface structure of nanoparticles, nanoparticles with rough surfaces and tip structures more easily undergo electromagnetic enhancement (EM) [11]. Noble metal nanoparticles represented by silver have efficient photoactivity and controllable morphology. Currently, Ag nanoparticles with different morphologies, such as nanocubes (NCs) and nanowires (NWs) [12,13], have been successfully synthesized. Among them, the flower-like Ag nanoparticles (F-Ag NPs) have a highly branched structure with rough particle surfaces and many tips, which can provide high-density electromagnetic “hotspots” [14]. Particles in a single noble metal colloid have the disadvantages of easy aggregation and poor stability, which inhibit the LSPR effect on the particle surface, leading to poor reproducibility and stability of SERS detection [15]. ZIF-8 is a typical MOF material with an abundant pore structure and excellent stability. When ZIF-8 is introduced, the prepared plasmonic MOF material has several advantages: (i) the problem of nanoparticle aggregation is greatly improved due to the introduction of porous materials, and the stability of the materials is improved at the same time [16,17]. (ii) The composites retain the LSPR properties of the noble metal particles while possessing the excellent porosity, tunability, and stability of MOFs. Due to the porous nature of MOFs, analyte molecules can be adsorbed on the plasma-activated surface, increasing the hotspot region and pre-enriching molecules at the hotspot location [18,19,20]. (iii) The synergistic effect of the two materials is able to enhance the localized surface electromagnetic field strength, achieving a substantial enhancement of the Raman signal of molecules near the surface [9,21]. Meanwhile, the hot electrons induced by the equipartitioned excitons can also modulate the chemical reactions of the surface molecules to improve the reaction rate and selectivity, i.e., the plasmon-mediated chemical reaction (PMCR) [22,23]. Despite the excellent SERS performance of plasmonic MOF materials, powder SERS substrates still suffer from separation difficulties and poor reproducibility.

Polyvinyl chloride (PVC) membranes have been widely used for antimicrobial and antifouling purposes as common flexible molecular membranes with high chemical stability, high flexibility, and low cost [24,25,26]. PVC membrane was chosen to prepare the flexible SERS substrate, which can provide support and protection for the powder material, improve the stability of the material, and increase the number of times the substrate can be reused [27]. Meanwhile, the preparation of a uniform membrane structure can avoid agglomeration or sparseness in the random arrangement of the substrate materials and improve the uniformity of the SERS signal. In addition, PVC is better suited for extreme testing environments, such as sewage and wastewater, due to its high resistance to chlorine, alkalis, and acids. Since the adsorption capacity of ZIF-8 is not selective, it will indiscriminately enrich molecules in the actual assay, which may interfere with the accuracy and detection efficiency of SERS [28]. In order to efficiently capture and detect target molecules in real samples and to increase the range of substrate applications, a new technique needs to be introduced for the specific recognition of target molecules.

Molecular imprinting technology (MIT) is a technique for molecular capture and specific recognition by preparing polymers that are highly matched to target molecules [29]. The predetermined, recognizable, and practical nature of the technique has led to its wide application in many fields (e.g., chromatographic separation [30], bionic sensing [31], clinical drug analysis [32], etc.). Methods exist to combine MIT with SERS for selective SERS detection [33]. Among them, the molecularly imprinted polymers prepared by precipitation polymerization were characterized by good dispersion and high yield [34,35]. Preparation of a layer of molecularly imprinted polymer on the surface of the SERS membrane can significantly enhance the selectivity and sensitivity of the assay. The imprinted polymer also protects the substrate material, increasing the lifetime of the substrate and reducing the limitations of the detection environment.

In this work, we used a chemical reduction method to prepare highly branched F-Ag NPs and synthesized ZIF-8 in situ on their surfaces to obtain plasmonic MOF materials with stable properties. The substrate helps to enrich the target and achieve spatial overlap between the target and the hotspot at the tip of the nanoparticle. Meanwhile, the synergistic effect of the two materials can enhance the localized surface electromagnetic field strength and, thus, the Raman signal. The optimal SERS performance was obtained by adjusting the reactant concentration to pre-enrich the target molecules closest to the hotspot. Considering the inherent drawbacks of powder materials, F-Ag@ZIF-8/PVC flexible substrates (FAZP) were prepared using the solvent-cast membrane method, which avoids the occurrence of agglomeration or thinning when the substrate materials are randomly arranged. PVC membranes were characterized by high mechanical strength and high resistance to chlorine, alkalis, and acids, which enhanced substrate stability and allowed them to be used for SERS testing even under extreme conditions. The prepared F-Ag@ZIF-8/PVC-MIM (FAZP-MIM) exhibited excellent sensitivity, selectivity, and stability for CG down to 10^−11^ M and showed excellent performance in the detection of real water samples.

## 2. Materials and Methods

### 2.1. Chemicals and Materials

Silver nitrate (AgNO_3_, ≥99%), 2-Methylimidazole (2-MI, 98%), ethylene glycol dimethacrylate (EGDMA, 98%), chrysoidin (CG), polyvinyl chloride (PVC), cyclohexanone (99.5%), and ascorbic acid (AA) were purchased from Aladdin Reagent (Shanghai, China). Zinc nitrate hexahydrate (Zn(NO_3_)_2_·6H_2_O, 98%) and acetic acid (CH_3_COOH) were purchased from Sinopharm Chemical Reagent Co., Ltd. (Shanghai, China). Polyvinylpyrrolidone (PVP, k30), ethanol (C_2_H_5_OH), methanol (CH_3_OH), acetonitrile, acrylamide (AM, 99%), 2-methylpropionitrile (AIBN, 98%), methylene blue (MB), methyl orange (MO), and Rhodamine B (RhB) were purchased from Tianjin Aopusheng Chemical Sales Co., Ltd. (Tianjin, China). Malachite green (MG) was purchased from Macklin Reagent (Shanghai, China).

### 2.2. Apparatus and Measurements

The micromorphology of the samples was obtained with a scanning electron microscope (JSM-7800 F, JEOL, Tokyo, Japan). The X-ray diffraction (XRD) spectra were collected on an X-ray diffractometer (PC2500, RIGAKU, Tokyo, Japan) with Cu Ka radiation over the 2θ range of 10–80°. Energy dispersive spectrograms (EDS) are determined by an energy dispersive spectrometer (EX-250, HORIBA, Kyoto, Japan). Infrared spectra (4000–400 cm^−1^) were obtained by a Fourier transform infrared spectrometer (IRAffinity-1S, Shimadzu, Kyoto, Japan). UV–Vis absorption spectra were determined by a UV–Vis spectrophotometer (UV-3600, Shimadzu, Kyoto, Japan). Raman spectra were recorded by a confocal micro-Raman spectroscopy system (SENTERRA II, RIGAKU, Tokyo, Japan).

### 2.3. Preparation of F-Ag NPs

Synthesis of F-Ag NPs was achieved by a chemical reduction method using AA as the reducing agent. Firstly, AgNO_3_ aqueous solution (0.06 M, 45 mL) and PVP aqueous solution (0.06 M, 45 mL) were added into a beaker, and after magnetic stirring for 30 min at room temperature, AA (0.2 M, 10 mL) was rapidly injected, and the solution immediately turned into dark grey color, which indicated that a large number of silver nanoparticles were formed. After magnetic stirring for 30 min, the reaction solution was centrifuged at 9500 rpm for 10 min, and then washed alternately with water and ethanol three times. Finally, the F-Ag NPs were dispersed in methanol and configured into a suspension of 0.025 M, which was used as a stock solution and kept in the refrigerator.

### 2.4. Preparation of F-Ag@ZIF-8

Synthesis of F-Ag@ZIF-8 by in situ generation of ZIF-8 on the surface of F-Ag NPs was carried out [36]. The synthesized 10 mL suspension of F-Ag NPs was put into a beaker. Then methanolic solution of Zn(NO_3_)_2_∙6H_2_O (0.025 M, 25 mL) and methanolic solution of 2-MI (0.2 M, 25 mL) were added successively under magnetic stirring for 2 h. After stirring for 2 h, the formed mixture was sealed and left to age for 24 h. The excess reactants were removed by washing several times with methanol and centrifuged to obtain F-Ag@ZIF-8. Different ratios of F-Ag@ZIF-8 were prepared by adjusting the amount of ZIF-8 added.

### 2.5. Preparation of F-Ag@ZIF-8/PVC

A total of 1.0 g of PVC was added to a beaker containing 5.0 mL of cyclohexanone and stirred magnetically to dissolve completely. Then, 0.2 g of F-Ag@ZIF-8 was added to the above membrane casting solution and stirred at room temperature for 6 h to make it completely dispersed in the mixed solution. The casting solution was poured onto a clean glass plate for scraping the membrane, and after complete evaporation of cyclohexanone, a yellow–grey membrane was obtained. The flexible membrane was cut to a size of 1 × 1 cm^2^ and stored in deionized water for spare use.

### 2.6. Preparation of F-Ag@ZIF-8/PVC-MIM

F-Ag@ZIF-8/PVC-MIM (FAZP-MIM) was prepared by precipitation polymerization. In 60 mL of acetonitrile, 10 pieces of flexible membrane and 0.8 mM AM were added, stirred well, and then 0.0248 g of CG, 2.4 mM of EGDMA, and 0.01 g of AIBN were added sequentially, and stirring was continued for 10 min. After the system was filled with enough N_2_ (15 min) to exclude air, the reaction was carried out at 50 ℃ for 6 h for pre-polymerization. Subsequently, polymerization was carried out by reacting the solution for 24 h at 60 °C. After the reaction, the blotting substrate was washed three times with ethanol and then further washed with methanol/acetic acid wash solution (*v*/*v* = 9/1) until the CG was completely removed. Finally, the obtained FAZP-MIM was placed in deionized water and set aside.

### 2.7. Raman Detection Measurement

Deionized water was used to configure different concentrations of CG solutions for assay use. For Raman detection, one piece of FAZP-MIM (1 × 1 cm^2^) substrate was placed in 20 mL of the corresponding concentration of CG standard solution for 12 h of static adsorption, removed and dried naturally. The FAZP-MIM substrate used for each experiment was prepared from the same batch. To ensure the accuracy of the results, each set of experiments was tested three times, and the average value was taken. In cyclic experiments, after each detection, the target molecules were removed with eluent, the adsorption–detection–elution process was repeated three times, and the Raman detection results of each process were recorded. In the actual sample testing, the known concentration of CG standard solution was added to the lake water and wastewater samples for spiked recovery detection, with other operations as above, and each concentration was repeated three times to calculate the actual recovery rate. To ensure the authenticity of the results, all SERS assays were performed under 532 nm laser excitation and the SERS spectra were acquired under a 50× OLYMPUS objective with an integration time of 10 s. The SERS spectra were obtained by using a 50× OLYMPUS objective with an integration time of 10 s.

## 3. Results and Discussion

### 3.1. Synthesis Process and Characterisation of FAZP-MIM

**Scheme 1:**
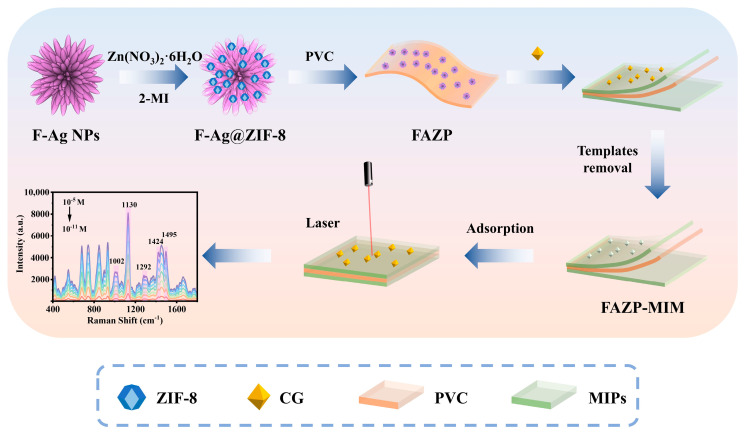
Schematic diagram of the preparation and detection process of FAZP-MIM.

In this study, FAZP-MIM was successfully prepared for the selective detection of CG. The FAZP-MIM preparation process was shown in Scheme 1. F-Ag NPs with a tip effect were synthesized via chemical reduction, followed by the successful synthesis of F-Ag@ZIF-8 by loading ZIF-8. Utilizing the adsorption properties of ZIF-8, molecules in the solution were enriched at the tip hotspots of the substrate material, significantly enhancing SERS performance. FAZP was prepared using a solvent casting method, creating a film material with good optical performance that protects and supports F-Ag@ZIF-8, facilitating its separation from the solution to be tested. The combined action of PVC and ZIF-8 significantly improved the stability and reusability of the substrate. Subsequently, the molecularly imprinted substrate FAZP-MIM was prepared using a precipitation polymerization method for the selective detection of CG.

**Figure 1:**
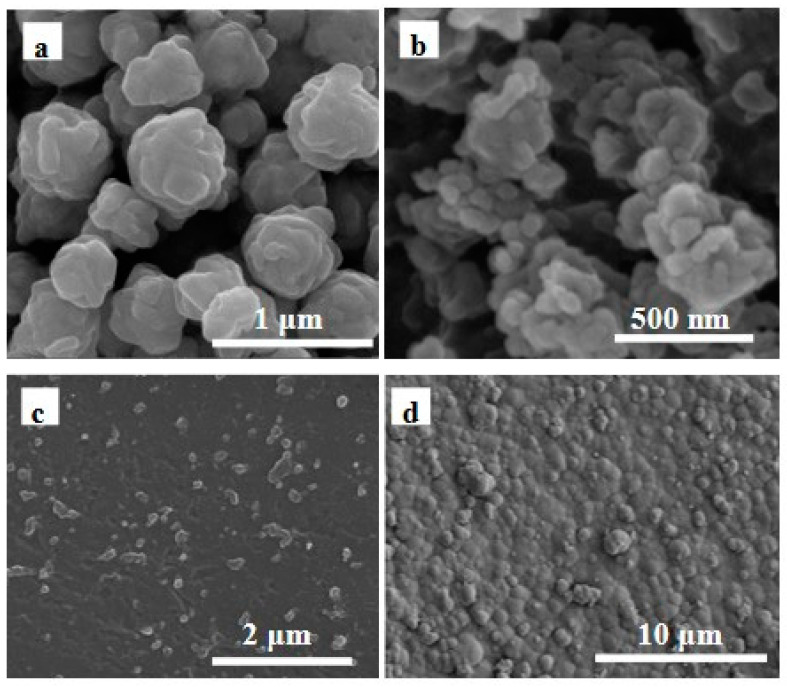
SEM images of (**a**) F-Ag NPs, (**b**) F-Ag@ZIF-8, (**c**) FAZP, and (**d**) FAZP-MIM.

The morphology of the substrate materials obtained during the synthesis of FAZP-MIM was characterized by SEM, as shown in Figure 1. The morphology of the F-Ag NPs prepared by the chemical reduction method was shown in Figure 1a. The particle size distribution of the particles was in the range of 500 nm–800 nm, and each F-Ag NP had a rough surface with the presence of many tips and gaps, which could provide abundant SERS hotspots. In Figure 1b, ZIF-8 uniformly loaded on the surface of F-Ag NPs with an average size of about 50 nm was observed. It was observed that ZIF-8 did not completely encapsulate the F-Ag NPs, and a certain gap between the particles was still retained, which was conducive to the enhancement of hotspots in the substrate material. Meanwhile, the high porosity of ZIF-8 was beneficial to the pre-enrichment of the detected molecules and favored the SERS sensitivity. To improve the uniformity and reproducibility of the substrate and prevent random arrangement and agglomeration of the powder substrate, high-quality optical PVC material was employed to create a membrane on F-Ag@ZIF-8. PVC possesses high flexibility and good chemical stability, along with a certain degree of resistance to chemical corrosion. Introducing PVC in the membrane preparation significantly protects the substrate material and enhances substrate stability. As shown in Figure 1c, the surface of the FAZP membrane material is uniform, with no signs of agglomeration. Subsequently, an imprinted polymer layer was prepared through precipitation polymerization, as shown in Figure 1d. It was observed that a uniform layer of polymer was added to the FAZP membrane surface, confirming the successful loading of the molecularly imprinted polymer.

**Figure 2:**
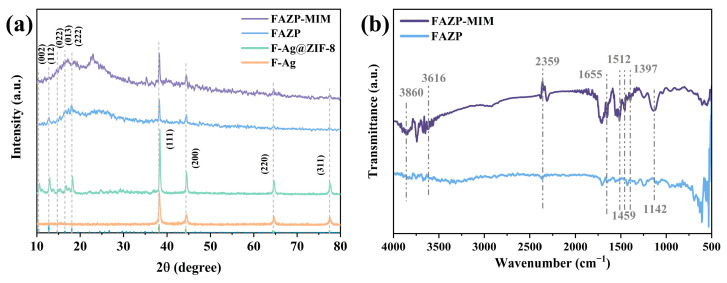
Different substrates of XRD and FT-IR characterization analysis. (**a**) XRD pattern presentation of F-Ag NPs, F-Ag@ZIF-8, FAZP, and FAZP-MIM; (**b**) FT-IR spectra of FAZP and FAZP-MIM.

Further characterization of the components and crystal structures of the synthesized F-Ag NPs, F-Ag@ZIF-8, FAZP, and FAZP-MIM was conducted using XRD. As shown in Figure 2a, sharp peaks appeared in the range of 2θ = 10–80°. The characteristic diffraction peaks at 2θ = 38.34°, 44.49°, 64.69°, and 77.66° corresponded to the face-centered cubic phase of Ag (JCPDS NO. 040783), corresponding to the Ag (111), (200), (220), and (311) planes. The crystallization peaks at 2θ = 10.55°, 12.98°, 15.09°, 16.77° and 18.29° were associated with the ZIF-8 polyhedron (002), (112), (022), (013), and (222) crystal planes, respectively [37]. It was clearly observed that after adding PVC and the imprinted layer, the intensity of the characteristic peaks decreased, but the lattice and structure of the F-Ag@ZIF-8 substrate remained unchanged. This confirmed that the substrate materials at each stage were successfully synthesized and that no structural damage occurred between them.

The FT-IR spectra of FAZP and FAZP-MIM were shown in Figure 2b, where the characteristic peak at 2359 cm^−1^ corresponds to the CO_2_ absorption peak during infrared testing. The peak at 1142 cm^−1^ was caused by the C-O-C stretching vibration in EGDMA, and the characteristic peak at 1397 cm^−1^ corresponded to the symmetric stretching vibration of -COO^−^. The peak at 3616 cm^−1^ was due to the O-H stretching vibration in -COOH, confirming that AM and EGDMA were successfully incorporated into FAZP. The characteristic peak at 1459 cm^−1^ corresponded to the symmetric vibration of C=O near the carboxyl group on the amino group of the imidazole ring. The characteristic peak at 1512 cm^−1^ corresponded to the C-O-Zn bond, and the characteristic peak at 1655 cm^−1^ corresponded to the bending vibration of N-H in the organic ligand [38,39]. These results collectively confirmed the successful preparation of FAZP-MIM.

The UV–vis spectra of the membrane materials before and after deposition polymerization of the imprinted layers were examined at room temperature. The UV–vis spectra of FAZP and FAZP-MIM are shown in Figure S1. It can be observed from the figure that the peak intensity increased overall after the deposition polymerization. The absorption peak at 221 nm was attributed to the charge transfer from the Zn-O group to the imidazole ring. The absorption band at 390 nm exhibited a red shift, mainly due to the functionalization of the N-H bond on the imidazole ring near the carboxyl group, facilitating charge transfer from Zn to the organic ligand [38,39,40]. The UV–vis results reflected the interactions of functional groups within the material, further confirming the successful preparation of the imprinted polymer.

### 3.2. Optimization of Synthesis Parameters

Firstly, different ratios of F-Ag@ZIF-8 were prepared by adjusting the content of ZIF-8 to initially modulate the substrate properties. The synthesis parameters of the three F-Ag@ZIF-8 were shown in Table S1. As shown in Figures S2 and S3, only a small amount of ZIF-8 was loaded on the F-Ag NPs when the content of ZIF-8 was added at a lower level, which had minimal impact on the Raman performance. When the quantity of reactants increased, ZIF-8 was evenly distributed on the F-Ag NPs with gaps, and the Raman signal was enhanced. When the quantity of reactants continued to increase, the over-synthesized ZIF-8 completely covered the surface of the F-Ag NPs without gaps, which hindered the identification of target analytes and reduced the Raman intensity, because only the F-Ag NPs had hotspots. Therefore, the second parameter was selected to synthesize F-Ag@ZIF-8 for subsequent experiments.

**Figure 3:**
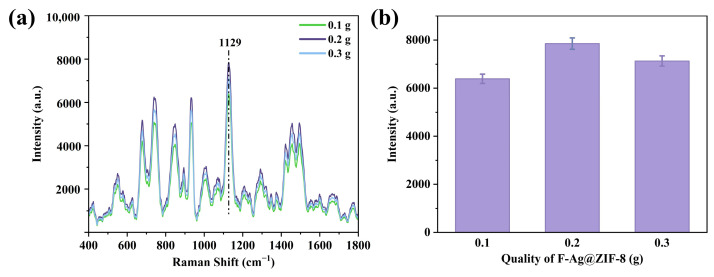
(**a**) SERS spectra of FAZP prepared with different contents of F-Ag@ZIF-8 against 10^−5^ M CG; (**b**) histogram of the intensity of characteristic peaks at 1129 cm^−1^.

To investigate the effect of the added F-Ag@ZIF-8 content on the Raman properties of FAZPs, FAZPs were prepared by adding 0.1 g, 0.2 g, and 0.3 g of F-Ag@ZIF-8, respectively, and the Raman properties of the corresponding FAZPs were tested against 10^−5^ M CG. From Figure 3, it can be seen that the Raman performance was lowest with the addition of 0.1 g F-Ag@ZIF-8, which is attributed to the lower content of F-Ag@ZIF-8 and the larger spacing between the hotspots. When 0.3 g of F-Ag@ZIF-8 was added, the Raman performance of the substrate was lower than that observed with the addition of 0.2 g. This was due to the agglomeration of excess F-Ag@ZIF-8, which hindered contact between the template molecule and the hotspot. At the same time, the excess powder substrate results in the suppression of the advantageous optical properties of PVC, leading to a degradation of Raman performance. Ultimately, 0.2 g of F-Ag@ZIF-8 was chosen for the preparation of FAZP for subsequent experiments.

### 3.3. Sensitivity of FAZP-MIM

The sensitivity of the SERS substrate had a decisive influence on practical applications. In order to compare the SERS performance of the substrates, the difference in sensitivity between FAZP and FAZP-MIM in CG solutions at higher (10^−5^ M) and lower (10^−11^ M) concentrations were analyzed, as shown in Figure S4. When the concentration of the target molecule was high, the sensitivities of detection of CG by FAZP and FAZP-MIM were similar, indicating that the Raman enhancement of SERS by the blotting layer was negligible at high concentrations. At this time, the target molecule was dominated by non-specific adsorption, primarily influenced by ZIF-8 in the plasmonic MOF material. Through the porous structure of ZIF-8, molecules were adsorbed to the hotspot regions, thereby providing the SERS signal. When the concentration of template molecules was low, it was difficult to capture molecules solely based on the adsorption capability of ZIF-8, and FAZP could hardly recognize the positions of the characteristic peaks. However, FAZP-MIM was still able to detect the presence of the characteristic peaks, which was related to the specific recognition sites of the template molecules on the imprinted layer. While ZIF-8 pre-concentrated the targets, the specific cavities on the imprint layer recognized the targets, capturing them in the hotspot regions. Under the synergistic effect of ZIF-8 and the imprint polymer, the concentration of target molecules at the SERS detection sites was higher, allowing the substrate to maintain excellent sensitivity even in low-concentration solutions.

**Figure 4:**
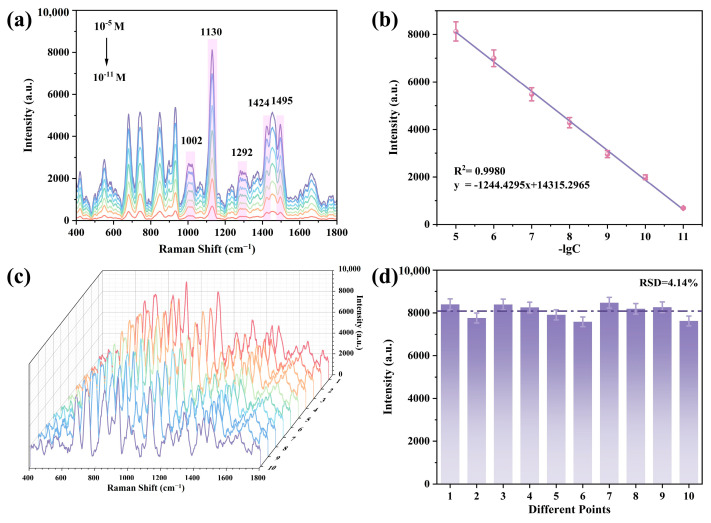
(**a**) SERS spectra of FAZP-MIM for different concentrations of CG solutions; (**b**) correlation curve between the intensity of the characteristic peak at 1130 cm^−1^ and the negative logarithm of CG concentration; (**c**) SERS spectra of 10 random sites on FAZP-MIM (10^−5^ M CG); (**d**) histogram of intensity of characteristic peaks at 1130 cm^−1^. The dashed line shows the mean value of SERS intensity obtained from ten SERS detections.

In order to further evaluate the sensitivity of FAZP-MIM for SERS detection of organic dye molecules, SERS was conducted on CG solutions with a concentration gradient (10^−5^ M–10^−11^ M) using FAZP-MIM and compared with the standard Raman spectra of CG. As shown in Figure S5, the standard Raman spectrum of CG indicates that the peak at 1001 cm^−1^ corresponds to the in-plane deformation vibration of the C-C bond, while the peak at 1147 cm^−1^ is attributed to the C-N bond stretching vibration and the N=N bond wobble vibration. The peak at 1294 cm^−1^ corresponds to the C-H bond wobble vibration and the N=N bond stretching vibration, whereas the peak at 1495 cm^−1^ relates to the stretching vibration of the C-C bond [41]. As can be seen from Figure 4a, the Raman spectra of CG obtained using FAZP-MIM exhibited characteristic peaks at 1002 cm^−1^, 1130 cm^−1^, 1292 cm^−1^, 1424 cm^−1^, and 1495 cm^−1^. The positions of the characteristic peaks on the substrate shifted compared to those of the CG characteristic peaks in Figure S5, which resulted from the interaction between the CG molecules and the metal nanoparticles on the substrate. The intensity of the characteristic peaks diminished with the decreasing CG concentration, with the lowest detected concentration being 10^−11^ M. As shown in Figure 4b, a strong linear relationship existed between the peak at 1130 cm^−1^ and the negative logarithm of the CG concentration, with an R^2^ value of 0.9980 for CG concentrations ranging from 10^−5^ M to 10^−11^ M. The results demonstrated that the FAZP-MIM substrate exhibited high sensitivity to the organic dye molecule CG, confirming its potential for practical quantitative detection.

The repeatability and uniformity of SERS signals represent key performance indicators of SERS substrates. The uniformity of the substrate was assessed by using FAZP-MIM for SERS of 10^−5^ M CG solutions, and 10 random sites were selected to extract Raman spectra. As shown in Figure 4c, the characteristic peaks at 1002 cm^−1^, 1130 cm^−1^, 1292 cm^−1^, 1424 cm^−1^, and 1495 cm^−1^ corresponded well, and the intensities of the characteristic peaks were similar with only minor fluctuations. The intensity of the characteristic peak at 1130 cm^−1^ was selected for histogram plotting, and the relative standard deviation (RSD) of the 10 random sites was 4.14%, as illustrated in Figure 4d. The data indicated that the prepared FAZP-MIM substrate had excellent reproducibility and that the substrate was highly uniform, which ensure the data’s credibility and detection efficiency in practical detection.

### 3.4. Selectivity of FAZP-MIM

**Figure 5:**
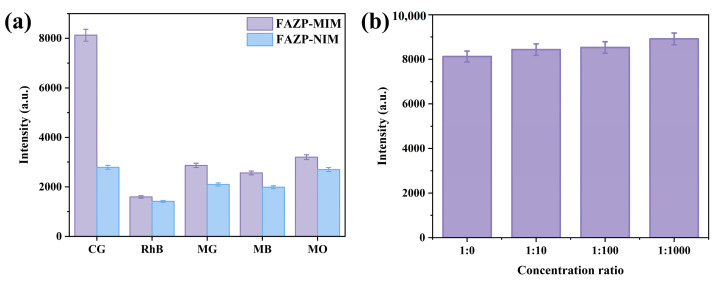
(**a**) SERS intensity histograms of five dyes (10^−5^ M) at the characteristic peak 1130 cm^−1^ detected using FAZP-MIM and FAZP-NIM substrates; (**b**) Raman intensities of mixed solutions at 1130 cm^−1^ for interfering molecule concentrations of 0, 10, 100, and 1000 times the concentration of CG.

Considering the complex environment of actual detection, two substrate materials, FAZP-MIM and FAZP-NIM, were employed to assess the selectivity of the FAZP-MIM substrate for CG among five organic dyes (CG, RhB, MG, MB, and MO). As shown in Figure 5a, the differences in SERS intensities for the five dyes on FAZP-NIM were not significant, with all intensities remaining low. The Raman signal observed on FAZP-NIM primarily results from non-specific adsorption of molecules on the substrate surface. In contrast, the FAZP-MIM substrate exhibited greater sensitivity to CG while demonstrating lower sensitivity to the other four dye molecules. This finding indicates that the molecularly imprinted cavities within FAZP-MIM substrates exhibit strong selectivity for CG, allowing for rapid adsorption and detection of template molecules in complex environments. To further investigate the specific recognition capability of FAZP-MIM for mixed solutions, the aforementioned dye molecules were mixed in various ratios and analyzed via SERS. During this test, the concentration of CG was maintained at 10^−5^ M (Figure 5b). When the concentration of the interfering molecules was 10, 100, and 1000 times that of CG, the Raman intensity at 1130 cm^−1^ increased by 3.82%, 4.98%, and 9.73% over the single CG signal, respectively. This phenomenon is hypothesized to occur because high concentrations of interfering molecules lead to ZIF-8 adsorption and non-specific adsorption on the substrate surface, resulting in molecular attachment that slightly affects the Raman signal. Nonetheless, the signal fluctuation within 10% across three orders of magnitude in concentration still demonstrates that the molecularly imprinted cavities within the FAZP-MIM substrate exhibit strong selectivity for CG, facilitating rapid adsorption and detection of template molecules in complex environments.

### 3.5. Stability of FAZP-MIM

**Figure 6:**
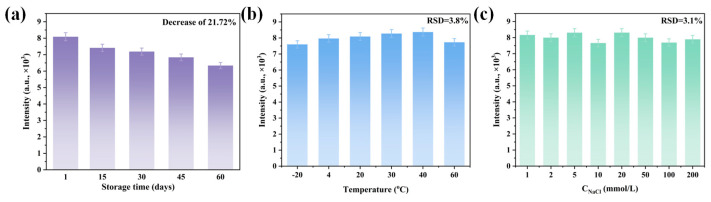
(**a**) SERS performance of FAZP-MIM with different storage times; (**b**) SERS performance of FAZP-MIM at different temperatures; (**c**) SERS performance of FAZP-MIM under different NaCl concentrations.

In a typical SERS analysis, the performance of the substrate can be affected by complex environments; therefore, the stability of the SERS substrate is crucial. In this experiment, the properties of ZIF-8 were utilized to enhance the stability of the silver nanoflowers. PVC, functioning as a flexible polymer membrane, demonstrates remarkable flexibility and chemical stability. The introduction of PVC enhances the substrate’s stability, enabling it to retain substantial SERS performance even in extreme detection conditions. The stability of FAZP-MIM was assessed under multiple conditions by monitoring the variations in the SERS intensity of CG on the substrate. After storing the FAZP-MIM substrate for 15, 30, 45, and 60 days, Raman detection was performed on 10^−5^ M CG solutions. As shown in Figure 6a, even after 60 days of storage, the substrate material still has good sensitivity, with only a 21.72% decrease in SERS intensity at 1130 cm^−1^ compared to the first day. More importantly, the stability of FAZP-MIM under different conditions of temperature and salt ion concentration was also investigated. As shown in Figure 6b, the substrate has good stability over a wide temperature range and detects 10^−5^ M CG with an RSD of 3.8%. The substrate was evaluated by modulating the salt ion concentration, and the RSD was 3.1% when the concentration of NaCl was in the range of 1 mM–200 mM (Figure 6c). Both ZIF-8 and PVC had a certain thermal and chemical stability, in which the stable topology of ZIF-8 protected the internal hotspot. The transparent flexible PVC membrane not only showed excellent optical performance, but also protected the internal plasmonic MOF material with excellent dirt and corrosion resistance. As a result, FAZP-MIM remained highly sensitive for a certain storage time and maintained SERS performance under extreme conditions, which helped to realize practical detection applications.

### 3.6. Recycling of FAZP-MIM

To test the cycling performance of the FAZP-MIM substrate, the substrate was sufficiently washed and then subjected to Raman detection again, which was repeated three times, and the Raman intensities of the three replicate experiments at the characteristic peak of 1130 cm^−1^ were compared. As shown in Figure S6a, the position of the characteristic peaks did not change in each cycle. The intensity of the characteristic peak gradually decreased with the increase in the number of cycles, and after three cycles, the intensity of the characteristic peak at 1130 cm^−1^ decreased to 93.1% of the initial intensity, as shown in Figure S6b. The decrease in Raman intensity can be attributed to the continued occupation of the cavity by a small number of target molecules, as well as the destruction of some of the cavity structures during elution, resulting in a lower adsorption capacity and a decrease in Raman intensity upon reuse. However, after three cycles, the Raman signal of CG was still above 90% of the initial intensity, indicating that the presence of the PVC membrane and the imprinted layer had a certain protective effect on the internal F-Ag@ZIF-8, and that FAZP-MIM still had a high level of Raman intensity after several cycles of washing, with excellent recycling properties.

### 3.7. Inspection of Actual Samples

**Figure 7:**
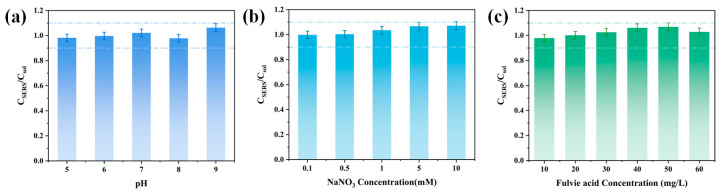
Effect of water conditions on FAZP-MIM as a SERS substrate for the detection of CG: (**a**) pH; (**b**) ionic strength; (**c**) humic acid concentration.

In order to realize the detection of CG in environmental water by FAZP-MIM, the effects of different water conditions on the detection of CG by FAZP-MIM as a substrate were first explored. Considering the abundance of nutrients in natural waters, three aspects were examined: pH, ion concentration and total organic carbon content. In this case, HCl and NaOH were used to adjust the solution pH, and the performance of the substrates was examined in the pH range of 5–9. Changes in ionic strength in aqueous environments were modelled by varying concentrations of NaNO_3_. In addition, since humic acid occupies 40–80% of the total organic carbon in natural waters, simulation experiments were conducted by adjusting the humic acid concentration. The detected SERS intensity was calculated from the linear relationship in Figure 4b to obtain the concentration of detection (C_SERS_). As shown in Figure 7, C_SERS_ had a good phenomenological correlation with the actual spiked concentration (C_sol_), and C_SERS_/C_sol_ was in the range of 0.9–1.1, which was satisfactory for the experimental results. These results provide preliminary evidence that FAZP-MIM substrate could be used for SERS detection of CG in environmental waters.

In order to evaluate the ability of the substrate to detect actual samples, SERS was performed on actual water samples. In this case, the experimental water samples were taken from Lake Denen and wastewater, and the insoluble impurities in the water samples were filtered and kept in the refrigerator for subsequent use. Recovery studies were carried out using Lake Denen water samples with detection concentrations of 1 × 10^−4^, 2 × 10^−4^, 3 × 10^−4^ M. The results of the study were shown in Table 1. The concentration of the target molecule could be calculated by measuring the Raman intensity. The recoveries of the samples in Lake Denen water samples were 95.96–109.50% with RSD < 4.5% (2.964–4.082%) in three replicates for each concentration. For industrial wastewater spiked with a CG concentration of 10^−4^ M, the resulting SERS spectrum is shown in Figure S7. The low Raman intensity detected in industrial wastewater may be due to the complex chemical composition of the wastewater and the excessive amount of interfering substances. Small molecular weight interfering substances would occupy the imprint cavity, while other interfering substances would be non-specifically adsorbed on the substrate surface due to the adsorption ability of ZIF-8, thus seriously affecting the accuracy of the Raman signal. Nevertheless, the characteristic peaks of CG were still clearly recognized, proving that the material had great potential to be applied to the detection of CG contaminants in real samples.

The performance of the method was further evaluated by comparing the data of the method with the performance parameters of published methods for detecting CG. Some of them are listed in Table S2 for comparison with our work. Compared to the literature reports, the present study has high sensitivity and selectivity, and its excellent stability makes it reusable. Thus, the substrate is capable of the sensitive and selective detection of trace CG and can be used as a reliable practical assay.

## 4. Conclusions

In conclusion, a F-Ag@ZIF-8/PVC-MIM flexible SERS blotting membrane for selective detection of CG was prepared in this study. The preparation of the plasmonic MOF material was achieved by loading ZIF-8 on the surface of F-Ag NPs with the tip effect. The synergistic interaction of ZIF-8 with highly branched F-Ag NPs was utilized to increase the number of hotspots, and the adsorption capacity of ZIF-8 allowed the target to be pre-enriched in the hotspot region. F-Ag@ZIF-8/PVC flexible SERS membranes with anti-fouling and anti-corrosion capabilities were prepared by adding PVC. FAZP-MIM was prepared in combination with molecular blotting technology, which provided selective recognition on the basis of molecular enrichment. FAZP-MIM has a high sensitivity with a detection limit as low as 10^−11^ M for CG. The SERS signal intensity at 1130 cm^−1^ showed a good linear relationship with the CG concentration in the range of 10^−5^–10^−11^ M, with a correlation coefficient (R^2^) of 0.9980. The imprinted layer was prepared uniformly with a relative standard deviation of 4.14%. Due to the properties of ZIF-8 and PVC, the prepared FAZP-MIM had desirable stability and maintained a high SERS performance under extreme conditions. It is also reusable, with the Raman intensity retaining 93.1% of its initial value even after three reuses. The substrate is highly selective for CG, and the method showed good recoveries of 96.98–104.75% in spiking and recovery tests on real lake water samples, and still identified clear CG spectra in industrial wastewater. In addition, due to the high sensitivity and stability of FAZP-MIM, the quantity of reagents used and the number of tests performed can be greatly reduced, thus lowering operating costs. This high efficiency and low cost means that FAZP-MIM has great potential for application in the detection of dyes in the environment.

## Figures and Tables

**Table 1:** Determination of CG in the lake water sample (n = 3).

Sample	Added (mol·L^−1^)	Found (mol·L^−1^)	Recovery (%)	RSD (%)
CG	1 × 10^−5^	1.095 × 10^−5^	109.50	4.082
CG	2 × 10^−5^	1.973 × 10^−5^	98.65	3.571
CG	3 × 10^−5^	2.879 × 10^−5^	95.96	2.964

## Data Availability

Data is unavailable due to privacy.

## Supplementary Materials

The following supporting information can be downloaded at: https://www.mdpi.com/article/10.3390/polym17010081/s1. Figure S1: UV-Vis spectra of FAZP and FAZP-MIM; Figure S2: SEM images of synthesised F-Ag@ZIF-8 with different ZIF-8 parameters (a) 1; (b) 2; (c) 3; Figure S3: Raman intensity of F-Ag@ZIF-8 prepared in different ratios; Figure S4: SERS spectra of different CG concentrations (10^−5^ M and 10^−11^ M) detected on FAZP and FAZP-MIM; Figure S5: Standard Raman spectrum of CG at 532 nm excitation wavelength; Figure S6: (a) SERS spectra obtained after three cycles of FAZP-MIM (10^−5^ M CG); (b) comparison of relative intensities of characteristic peaks at 1130 cm^−1^; Figure S7: SERS spectra of FAZP-MIM on 10^−4^ M CG in wastewater samples; Table S1: Specific variables and parameters for F-Ag@ZIF-8 preparation; Table S2: The comparison between present method and recent literature. References [3,41,42,43,44] are cited in the supplementary materials.
