# Supplementary material for: Highly Stable Flexible SERS-Imprinted Membrane Based on Plasmonic MOF Material for the Selective Detection of Chrysoidin in Environmental Water
Source: Polymers (Basel). 2024 Dec 31;17(1):81. doi: 10.3390/polym17010081 (PMC11723279; doi:10.3390/polym17010081)
Supplement: Supplementary file 1 [file polymers-17-00081-s001.zip › polymers-3312707-supplementary.pdf]

Supplementary Material

Highly Stable Flexible SERS-Imprinted Membrane Based on Plasmonic MOF Material for the Selective Detection of Chrysoidin in Environmental Water

Xinyi Liu [1], Hongji Li [1,*], Dandan Wang [1,2,*], Jian Lu [3], Yilin Wu [4] and Wei Sun [1]

[1] Hainan Engineering Research Center of Tropical Ocean Advanced Opto-electrical Functional Materials, College of Chemistry and Chemical Engineerin, Hainan Normal University, Haikou 571158, China
[2] Key Laboratory of Advanced Materials of Tropical Island Resources, Ministry of Education, School of Chemistry and Chemical Engineering, Hainan University, Haikou 570228, China
[3] School of Fashion and Textiles, The Hong Kong Polytechnic University, Hong Kong 999077, China.
[4] Institute of Green Chemistry and Chemical Technology, Advanced Chemical Engineering Laboratory of Green Materials and Energy of Jiangsu Province, School of Chemistry and Chemical Engineering, Jiangsu University, Zhenjiang 212013, China.
* Correspondence: H.J. Li: hongjili1102@hainnu.edu.cn
D.D.Wang: 184499@hainanu.edu.cn

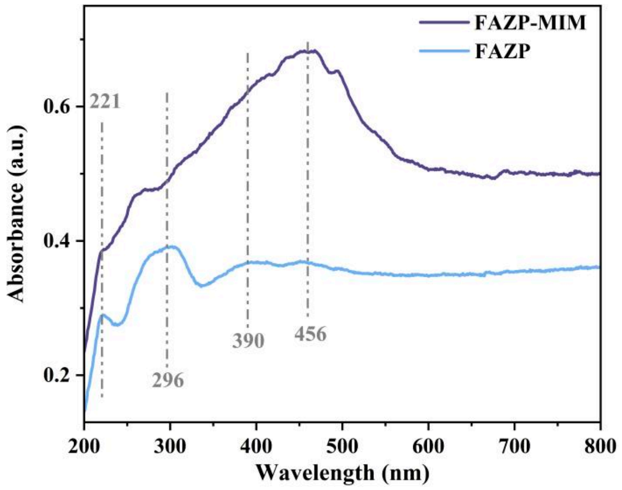

Figure S1. UV-Vis spectra of FAZP and FAZP-MIM.

Table S1. Specific variables and parameters for F-Ag@ZIF-8 preparation.

F-Ag@ZIF-8	F-Ag NPs (mL)	$Zn(NO_3)_2{\cdot}6H_2O$ (g/mL)	2-MI (g/mL)
**1**	10	3.72×10^{-3}	8.21×10^{-3}
**2**	10	7.44×10^{-3}	16.42×10^{-3}
**3**	10	14.87×10^{-3}	32.83×10^{-3}

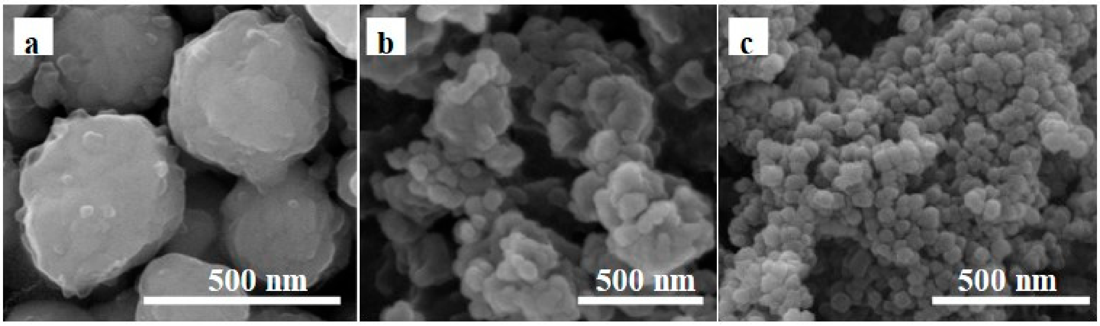

Figure S2. SEM images of synthesised F-Ag@ZIF-8 with different ZIF-8 parameters (**a**) 1; (**b**) 2; (**c**) 3.

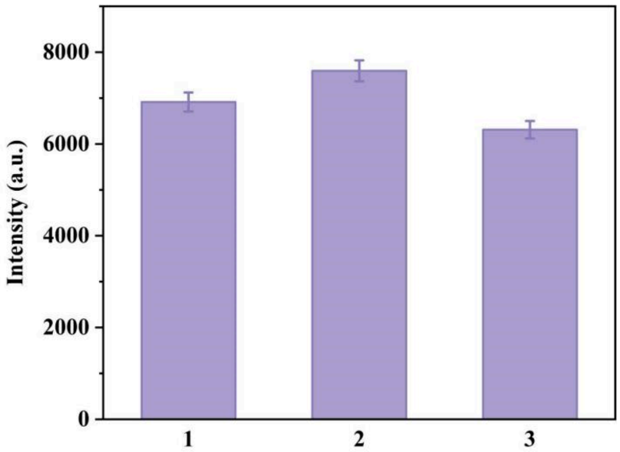

Figure S3. Raman intensity of F-Ag@ZIF-8 prepared in different ratios.

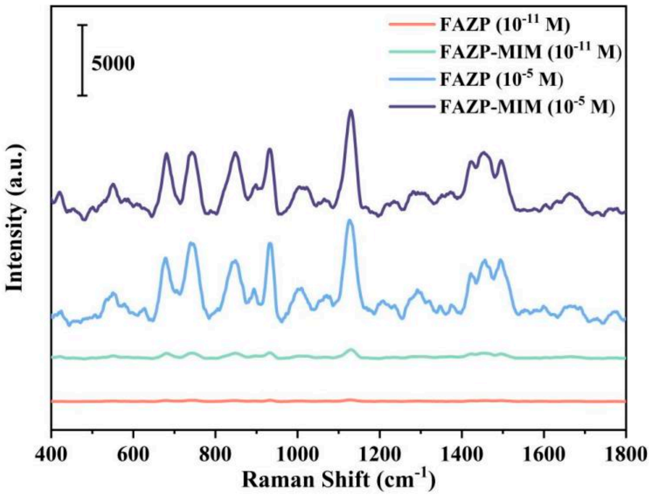

Figure S4. SERS spectra of different CG concentrations (10^{-5} M and 10^{-11} M) detected on FAZP and FAZP-MIM.

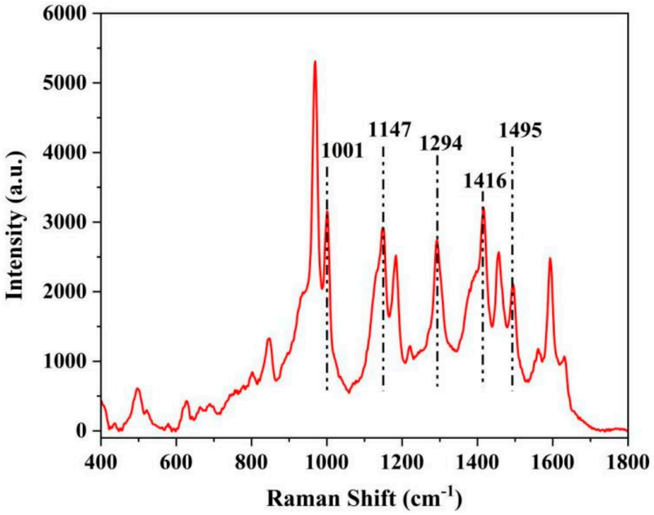

Figure S5. Standard Raman spectrum of CG at 532 nm excitation wavelength.

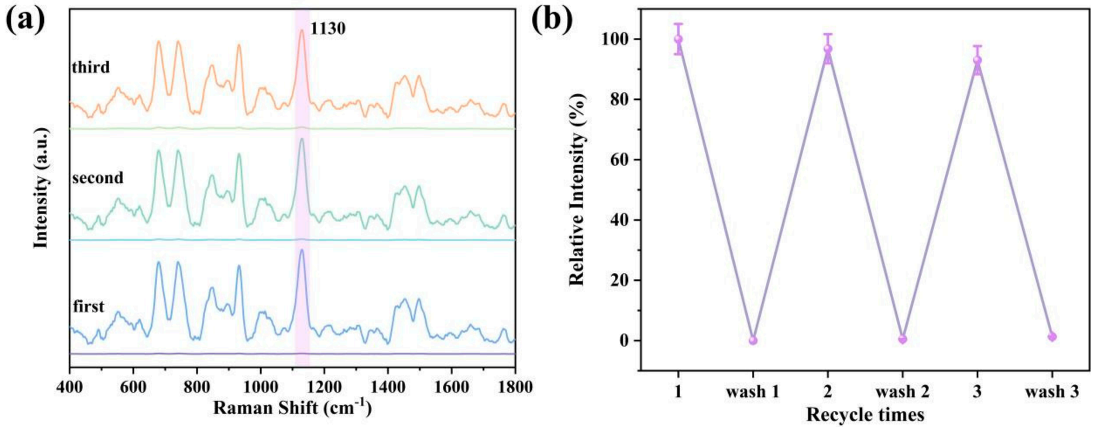

Figure S6. (**a**) SERS spectra obtained after three cycles of FAZP-MIM (10^{-5} M CG); (**b**) comparison of relative intensities of characteristic peaks at 1130 cm^{-1}.

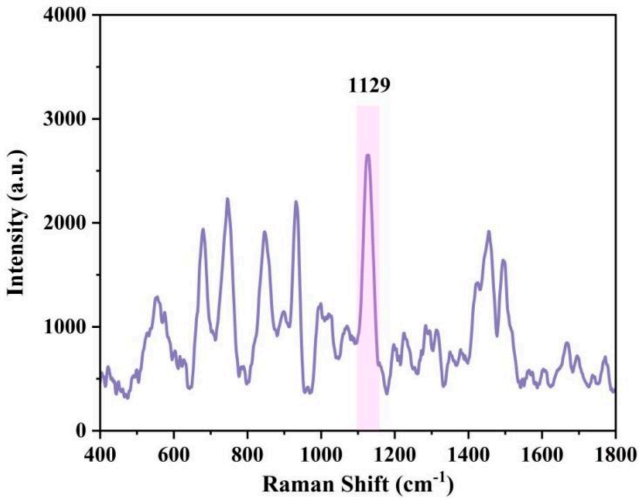

Figure S7. SERS spectra of FAZP-MIM on 10^{-4} M CG in wastewater samples.

Table S2. The comparison between present method and recent literature.

Detection Methods	Working Range	LOD	Selectivity	Reusability	Reference
solid-phase extraction	0.5–11 μg/mL	4×10^{-8} M	Yes	/	[1]
solid-phase extraction	0.5–0.05 μg/mL	2.01×10^{-7} M	Yes	/	[2]
SERS	10^{-6}–10^{-9} M	10^{-9} M	No	No	[3]
SERS	0.05–0.001 mg/L	4×10^{-9} M	No	No	[4]
SERS	10–0.5 μg/mL	2.01×10^{-6} M	No	No	[5]
This work	10^{-5}–10^{-11} M	10^{-11} M	Yes	Yes	This work

References

[1] X. Li, M. Li, J. Li, F. Lei, X. Su, M. Liu, P. Li, X. Tan, Synthesis and characterization of molecularly imprinted polymers with modified rosin as a cross-linker and selective SPE-HPLC detection of basic orange II in foods. Analytical Methods. 2014, 6, 6397.

[2] W. Sun, Q. Xu, Q. Liu, T. Wang, Z. Liu, Post-synthetic modification of a magnetic covalent organic framework with alkyne linkages for efficient magnetic solid-phase extraction and determination of trace basic orange II in food samples. Journal of Chromatography A. 2023,1690, 463777-463777.

[3] B. Zhao, R. Hao, Z. Wang, H. Zhang, Y. Hao, C. Zhang, Y. Liu, Green synthesis of multi-dimensional plasmonic coupling structures: Graphene oxide gapped gold nanostars for highly intensified surface enhanced Raman scattering. Chemical Engineering Journal. 2018, 349, 581-587.

[4] Z. Zhang, L. Sun, Y. Zhang, Y. Kang, H. Hu, J. Iqbal, Y. Du, Rapid determination of illegal additives chrysoidin and malachite green by surface-enhanced Raman scattering with silanized support based substrate. Chinese Chemical Letters. 2018, 29, 981-984.
[5] Y. Sun, W. Li, L. Zhao, F. Li, Y. Xie, W. Yao, W. Liu, Z. Lin; Simultaneous SERS detection of illegal food additives rhodamine B and basic orange II based on Au nanorod-incorporated melamine foam. Food Chem. 2021, 357, 129741.
